# Development and validation of a guideline on sexual and reproductive health of breast cancer survivors in Iran: a mixed methods study protocol

**DOI:** 10.1186/s12961-021-00738-6

**Published:** 2021-05-31

**Authors:** Solmaz Roshandel, Minoor Lamyian, Seyed Ali Azin, Shahpar Haghighat, Eesa Mohammadi

**Affiliations:** 1 Department of Reproductive Health and Midwifery, Faculty of Medical Sciences, Tarbiat Modares University, Tehran, Iran; 2 Reproductive Biotechnology Research Center, Avicenna Research Institute, ACECR, Tehran, Iran; 3 Breast Cancer Research Center, Motamed Cancer Institute, ACECR, Tehran, Iran; 4 Department of Nursing, Faculty of Medical Sciences, Tarbiat Modares University, Tehran, Iran

**Keywords:** Sexual and reproductive health, Breast cancer, Clinical practice guideline, Mixed methods study, Study protocol

## Abstract

**Background:**

Sexual and reproductive health problems significantly decrease quality of life in survivors of breast cancer. The best approach is to provide services according to evidence-based guidelines developed based on their practical context. Here, we aim to develop and validate a guideline on the sexual and reproductive health of breast cancer survivors in Iran.

**Methods:**

The guideline will be developed and validated using an exploratory sequential mixed methods approach in three phases: (1) describing sexual and reproductive health needs of survivors of breast cancer in Iran and the health services they receive in this regard, (2) performing a systematic review of existing guidelines, resources, and documents on the sexual and reproductive health of breast cancer survivors worldwide, and (3) developing and validating a guideline on the sexual and reproductive health of women who survived breast cancer in Iran based on the results of phases 1 and 2 through multiple steps.

**Discussion:**

A comprehensive and practical guideline on the sexual and reproductive health of breast cancer survivors in Iran will be developed which will be compatible with their specific needs and culture, considering the limited resources available. This guideline can significantly improve the quality of life in breast cancer survivors in Iran. In addition, the approach we will use here can be utilized to develop guidelines on sexual and reproductive health of female cancer survivors in general.

## Background

Breast cancer is the most common cancer in women in most countries around the world [1]. In Iran, breast cancer is not only the most common cancer in women, but also the most common cancer in the whole population regardless of sex [2]. Patients are typically diagnosed when they are in their 40s or 50s, with the mean age at diagnosis being 49 years. The age at diagnosis is approximately 10 years younger than the global average [3]. The number of breast cancer survivors is also increasing due to early diagnoses and advanced treatments. The 5-year survival rate is greater than 90% in the United States [4] and is 72% in Iran [5].

Breast cancer survivors experience sexual and reproductive health problems [4, 6]. After breast cancer diagnosis and treatment, all aspects of sexual function including libido, arousal, and orgasm are often deteriorated. Patients experience dyspareunia and negative mental experiences such as negative body image, loss of femininity, concerns about infertility, lack of sexual attractiveness, depression, and anxiety, leading to reduced sexual satisfaction and finally discontinuing sexual relations [6, 7]. For example, a study in Iran showed that sexual dysfunction is more common in breast cancer survivors than in healthy women. They experience dyspareunia and problems in sexual desire, vaginal lubrication, arousal, and orgasm [8]. Sexual function also deteriorates over time in many patients. Young age, hormone therapy, and poor sexual function at the time of diagnosis are the main factors associated with worse sexual function [9]. Often, sexual health is not the priority. Both patients and therapists are hesitant to talk about sexual problems due to embarrassment and lack of privacy, time, or required skills [10, 11].

In addition, many cancer treatments can cause infertility or premature menopause [12]. Infertility can be very stressful, especially in women with no children [13]. Although there are currently various solutions to preserve fertility, they may not all be practical [12]. Discussing concerns about infertility with reproductive health specialists can reduce patients’ levels of stress, educate them about available options, and help them to decide how to preserve their fertility [14]. On the other hand, due to the teratogenic effects of cancer treatments, contraception should be considered during therapy, and young patients should receive required information immediately after diagnosis [15, 16].

Valid clinical guidelines are required to provide appropriate evidence-based care. Clinical guidelines should be carefully developed through an explicit process considering scientific evidence, health and medical care providers’ experiences, and patients’ needs. These guidelines can aid both physicians and patients in making complex medical decisions, leading to higher quality of treatment and better outcomes [17]. Clinical guidelines should also be developed according to the context in which they are intended to be used. Developing a new guideline requires access to extensive resources. To save resources and to avoid duplication of efforts, a combination of different approaches should be utilized, including adopting recommendations from existing guidelines as they are, adapting them by making changes to existing recommendations considering local circumstances, or providing new evidence-based recommendations if required [18].

Globally, a few guidelines are available for cancer survivor care, although they may not be specific to breast cancer or sexual and reproductive health. The American Cancer Society (ACS) and American Society of Clinical Oncology (ASCO) published the “American Cancer Society/American Society of Clinical Oncology Breast Cancer Survivorship Care Guideline” in 2015. Some parts of this guideline address sexual and reproductive health such as body image, sexual function, infertility, premature menopause, and hot flashes [19]. The European School of Oncology (ESO) and European Society for Medical Oncology (ESMO) also developed a guideline for young women with breast cancer, updated in 2018, entitled “ESO–ESMO 4th International Consensus Guidelines for Breast Cancer in Young Women (BCY4)”. This guideline also includes sexual and reproductive health [15]. Cancer Care Ontario (CCO) published a guideline in 2016, “Interventions to address sexual problems in people with cancer”, including recommendations for all adult male and female survivors of various cancers and their sexual partners. Parts of this guideline is specific to women with breast cancer [20]. In 2016, the National Comprehensive Cancer Network (NCCN) also published a guideline entitled “Sexual Function in Cancer Survivors” addressing issues related to the sexual health of survivors of various cancers [21]. The ASCO has a guideline as well, entitled “Fertility Preservation in Patients with Cancer” regarding preserving fertility in cancer survivors including breast cancer [22]. However, to our knowledge, there has been no guideline developed in Iran for the sexual and reproductive health of cancer survivors in general or breast cancer specifically. A comprehensive guideline based on scientific evidence considering local and cultural circumstances could significantly improve quality of life in these patients.

## Aim

Here, we aim to develop and validate a comprehensive guideline for sexual and reproductive health of breast cancer survivors in Iran, taking into account local and cultural needs and circumstances.

## Methods

### Study design

**Fig. 1:**
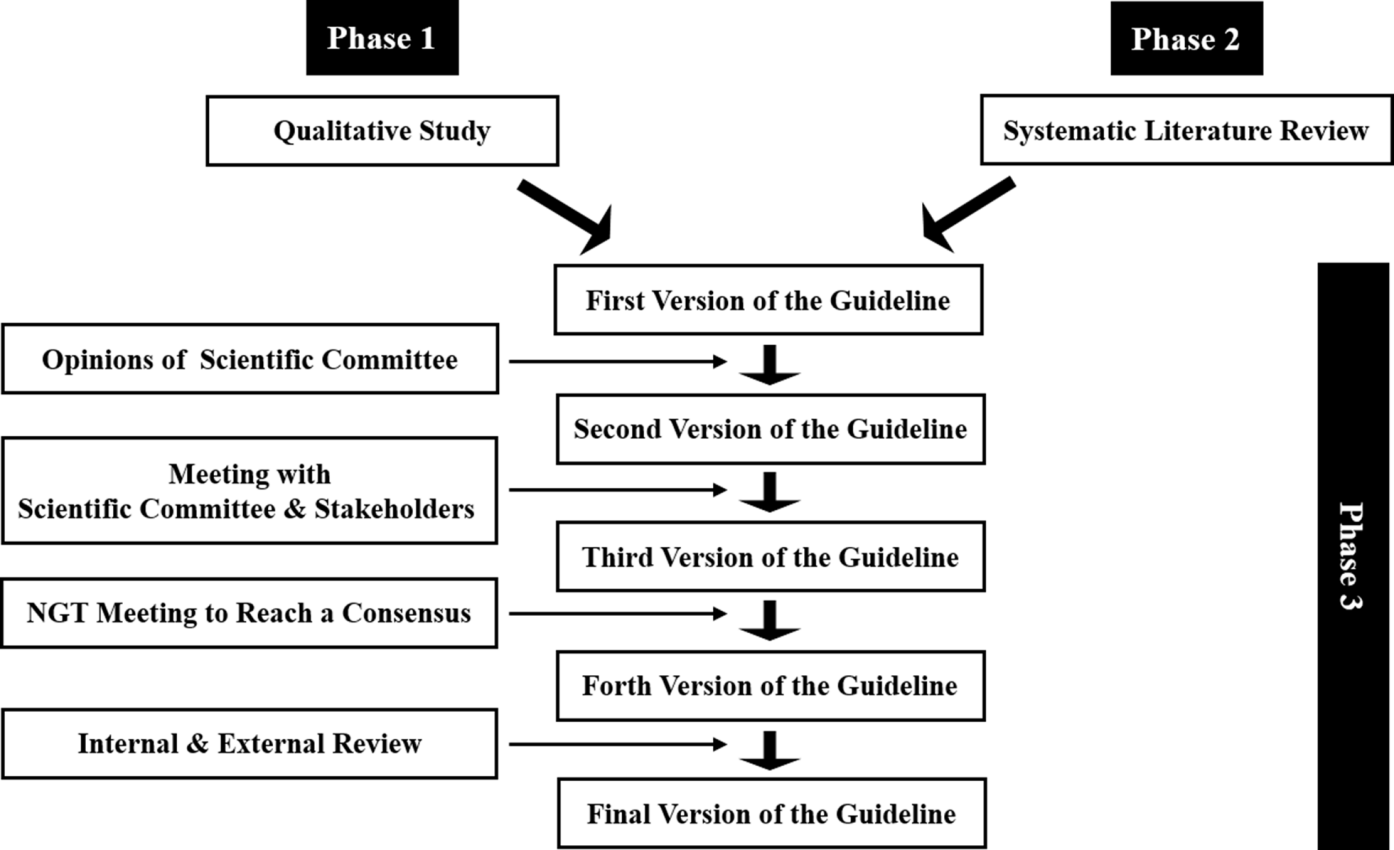
Study design. *NGT* nominal group technique

We will use an exploratory sequential mixed methods approach, starting with a qualitative data collection and analysis, followed by a quantitative data collection and analysis, and finally combining data from both [23]. The study will include three phases (Fig. 1).

In phase 1, we will collect qualitative data to explore breast cancer survivors’ sexual and reproductive health needs and the health services they receive in this regard. During this phase, we will identify which services are available in Iran, and whether breast cancer survivors have any difficulties accessing these services. We will also gain a profound understanding of the needs, preferences, and values of Iranian patients, their husbands, and healthcare professionals regarding sexual and reproductive health services. These data will be extremely useful for developing a guideline suitable for Iranian patients considering their local and cultural needs. Data will be collected through in-depth semi-structured interviews with women who have survived breast cancer, their husbands, and also health and medical service providers. We will use purposeful sampling (maximum variation) to maximize diversity. Sampling will continue until data saturation. Data will be analysed simultaneously using a conventional qualitative content analysis approach [24].

In phase 2, we will conduct a systematic review of all available guidelines, documents, and published papers on sexual and reproductive health in survivors of breast cancer or other cancers worldwide.

In phase 3, first, we will draft the initial version of the guideline using the results from phases 1 and 2. Subsequently, the guideline will be modified, validated, and finalized through multiple steps.

The guideline will be developed at the national level, and Iranian breast cancer survivors will be the target population. The users of the guideline will include all healthcare professionals providing services to breast cancer survivors at different levels (e.g. surgeons, oncologists, radiotherapists, obstetricians, infertility specialists, reproductive health specialists, psychologists, psychiatrists, counsellors, nurses, and midwives).

Multiple groups will be involved in the guideline development including the guideline development core group, the scientific committee, the oversight committee, and stakeholders.

The guideline development core group will include a health education expert (ML), a sexual health specialist (SAA), an epidemiologist (SH), a methodologist (EM), and a reproductive health PhD candidate (SR).

The scientific committee will include Iranian physicians with different specialties (i.e. surgeons, oncologists, obstetricians, infertility specialists, reproductive health specialists, and psychologists), psychiatrists, nurses, and midwives. The name and affiliation of the scientific committee and their conflicts of interest will be declared in the final version of the guideline.

The oversight committee will include university professors from Tarbiat Modares University, Tehran, Iran, and university professors from the other Iranian medical universities. They will supervise the process of guideline development. The name and affiliation of the oversight committee will also be declared in the final version of the guideline.

The stakeholders will include breast cancer survivors, their husbands and families, their healthcare providers, policy-makers, health system administrators, and insurance companies.

This study was approved by the ethics committee of Tarbiat Modares University, Tehran, Iran (IR. MODARES. REC. 1397.207). The details of each phase are presented in the next section.

### Phase 1: Qualitative study

#### Participants

The participants will include three groups: breast cancer survivors (women) at different stages of the disease (diagnosis, treatment, and follow-up), their husbands, and service providers with different levels of expertise.

We will use purposeful sampling (maximum variation) for recruiting breast cancer survivors to maximize diversity in terms of age (peri- or postmenopausal), marital status (single, married, widowed, or divorced), cancer stage, disease duration, and course of disease (diagnosis to follow-up). All participants (women with breast cancer and their husbands) should be willing to participate in the study, have Iranian nationality, can speak Farsi, and will be able to communicate to share their experiences.

The service providers will be selected from a wide range of people providing health and medical services to women with breast cancer including surgeons, oncologists, radiotherapists, nurses, midwives, obstetricians, infertility specialists, reproductive health specialists, counsellors, psychologists, psychiatrists, and follow-up personnel. They should have at least 2 years of related work experience in the public, private, or semi-private sector.

#### Study setting

Participants will be recruited from public, semi-private, and private centres providing services to patients with breast cancer in Tehran. The time and place of the interview will be arranged considering the participants’ choices.

#### Data collection

The code of ethics will be reviewed and required permissions will be obtained in advance at each centre. Eligible subjects will be informed about the research objectives and ethical considerations including confidentiality, no interference with the services they receive, destroying their audio files after completing the research, anonymity of the reports, and reflecting their views with honesty and no censorship. Subsequently, written informed consent will be obtained to record the interview and arrangements will be made in terms of the time and place of the interview. If the participant does not consent to record the interview, only notes will be taken.

The interviews with patients and service providers will be conducted by SR (PhD candidate, female). The interviews with husbands will be conducted by one of the male physicians within the guideline development core group (SAA), since Iranian men are more comfortable sharing their views with men due to Persian culture. The interviews will be semi-structured and expanded based on the participants’ replies to the initial general questions. General questions for breast cancer survivors, their husbands, and service providers are summarized in Table 1. The audio files will be transcribed and analysed by SR as soon as possible following the interview. The interviews will continue until data saturation when no new information or codes are recorded.

**Table 1:** General questions for breast cancer survivors, their husbands, and service providers

Individual	Questions
Breast cancer survivors	1. What sexual and reproductive health problems have you experienced after breast cancer diagnosis? 2. What concerns you in this regard? 3. Have you shared your concerns with any professional(s)? 4. What health and medical services have you received?
Brest cancer survivors’ husbands	1. What has changed in your marriage and sexual life after your wife was diagnosed with breast cancer? 2. How have you tried to solve the problems? 3. What health and medical services have you received?
Service providers	1. What sexual and reproductive health problems do breast cancer survivors have? 2. What kind of health and medical services do they receive in this regard?

#### Data analysis

The data will be analysed using a qualitative content analysis method as described by Graneheim and Lundman [24]. With this method, the entire interview is considered as the unit of analysis. Meaning units are words, sentences, or paragraphs containing related aspects. The meaning units are then condensed based on their manifest (a description close to the text) or latent (an interpretation of the underlying meaning) content. Subsequently, the condensed text is abstracted, and codes, categories, and themes are created on varying levels [24].

#### Establishing rigor and trustworthiness

We will ensure that the data are trustworthy, described by credibility, dependability, confirmability, transferability, and authenticity [25]. We will use different strategies to achieve credibility including prolonged engagement, member checking, and maximum diversity sampling. We will spend sufficient time for each interview and also for processing them. The processed text from some interviews will be returned to the participants to evaluate their consistency. In addition, as mentioned above, we will use purposeful sampling (maximum variation) to maximize the diversity and capture a wide range of perspectives. Data collection will continue until data saturation, and we will use multiple methods to collect the data including interviews, field notes, and investigating all documents to increase the validity of the data. To achieve confirmability, we will present all interviews and extracted codes and themes to the experts within the study group as well as experts outside the study group who are interested in this field for further investigation and confirmation. To increase dependability, in addition to member checking, everything will be well documented and will be accessible. To increase transferability, a clear and detailed description of the context, participant selection process and characteristics, and data collection and analysis will be provided so that other researchers can decide whether the findings can be transferred to their own settings. Constant observation, prolonged engagement, field notes, and detailed and comprehensive reporting will maximize authenticity.

### Phase 2: Systematic review of available guidelines worldwide

SR and one other member of the guideline development core group will independently conduct a comprehensive literature review to find all relevant guidelines, documents, and papers published in English or Farsi since 1 January 2000 in medical literature databases, clinical guideline databases, international cancer-related websites, and national medical databases summarized in Table 2. The search strategy is summarized in Table 3. We will combine terms within each category by “OR” and between categories by “AND”. Medical Subject Heading (MeSH) terms will be used only for searching in PubMed, and the other terms will be used in all databases.

**Table 2:** The databases and websites searched in phase 2

Category	Database	URL
Medical literature databases	PubMed	https://www.pubmed.ncbi.nlm.nih.gov/
	Scopus	https://www.scopus.com/home.uri
	Web of Science	https://www.webofknowledge.com
	Cochrane Library	https://www.cochranelibrary.com/
	ScienceDirect	https://www.sciencedirect.com/
	ProQuest	https://www.proquest.com
	CINAHL	https://www.ebscohost.com
	Trip Database	https://www.tripdatabase.com/
	UpToDate	https://www.uptodate.com/
Clinical guideline databases	Guidelines International Network (GIN)	https://www.g-i-n.net
	National Institute for Clinical Excellence (NICE)	https://www.nice.org.uk/Guidance/
	ClinicalKey	https://www.clinicalkey.com
	Scottish Intercollegiate Guidelines Network (SIGN)	https://www.sign.ac.uk/
	Agency for Healthcare Research and Quality (AHRQ)	https://www.ahrq.gov
International cancer-related websites	American Society of Clinical Oncology (ASCO)	https://www.asco.org/
	NCCN	https://www.nccn.org
	CCO	https://www.cancercareontario.ca
National medical databases	Scientific Information Database (SID)	https://www.sid.ir
	Magiran	https://www.magiran.com/
	Civilica	https://www.en.civilica.com/

**Table 3:** The literature search strategy

Category	Term
Guideline	“Practice guidelines as topic” [MeSH] “Guidelines as topic” [MeSH] Practice guideline Guidelin* Program Intervention
Sexual and reproductive health	“Sexual health” [MeSH] “Sexuality” [MeSH] “Reproductive health” [MeSH] “Reproductive health services” [MeSH] “Fertility” [MeSH] “Fertility preservation” [MeSH] “Contraception” [MeSH] “Infertility” [MeSH] “Reproduction” [MeSH] “Sexual health” Sexuality “Sexual function” “Sexual health services” “Sexual dysfunction” “Reproductive health” “Reproductive health services” Fertility “Fertility preservation”
Breast cancer	“Breast neoplasms” [MeSH] Cancer Neoplasm “Breast cancer” “Breast neoplasms” Survivor*

“OR” will be applied between terms within one category, and “AND” will be applied between categories

MeSH terms will be used in PubMed only, and the other terms will be applied in all databases

Subsequently, SR and one other member of the guideline development core group will screen available guidelines in English based on being up-to-date and validity of their source. In case of disagreement, they will consult one of the senior members of the guideline development core group. The screened guidelines will then be evaluated with the Appraisal of Guidelines, Research and Evaluation II (AGREE II) instrument [26].

AGREE II contains 23 items in six domains: scope and purpose, stakeholder involvement, rigor of development, clarity of presentation, applicability, and editorial independence. Each item is rated on a 7-point scale from 1 (strongly disagree) to 7 (strongly agree). Under each item, there is a space for further comments. A score is calculated independently for each of the six domains and is not accrued into a single score. Domain scores are useful for evaluating the quality of the guidelines, but there is no cut-off for classifying high- versus poor-quality guidelines. At the end, individuals will evaluate the guideline overall and will be asked whether or not they would recommend it. It is recommended that at least two and preferably four people assess the guidelines. AGREE II can be used by different groups: service providers who wants to use a guideline, guideline developers for self-evaluation, and policy-makers to decide which guideline would work best [26, 27]. AGREE II has been translated and used by Iranian researchers in the past [28].

Guidelines with a score greater than 40% in domain 3 of AGREE II (rigor of development) will be selected. Selected guidelines will be summarized in a table with their title, source, date of last update, and target audience. Recommendations regarding prevention, screening, diagnosis, and management of sexual and reproductive health problems will be included in the table.

The Grading of Recommendations Assessment, Development and Evaluation (GRADE) framework will be used to evaluate the recommendations [29]. The level of evidence for each recommendation will be assessed using information from the original guideline according to Table 4.

**Table 4:** Level of evidence

Level	Definition
1: Strong	Randomized clinical trials or their meta-analysis
2: Intermediate	Non-randomized clinical trials and their meta-analysis Case–control studies or their meta analysis Prospective cohort studies
3: Weak	Cross-sectional studies, observational studies, case series, or case reports
4: No evidence	Insufficient evidence

Adapted [38] from American Association of Clinical Endocrinologists (AACE) protocol for standardized production of clinical practice guidelines [39]

We will also incorporate recommendations that were not included in the guidelines but were proposed in peer-reviewed published papers after assessment of their quality.

### Phase 3: Guideline development and validation

First, we will use the PIPOH technique to develop a set of key questions [30]. This technique includes five items: Population concerned (survivors of breast cancer in Iran), Interventions of interest (sexual and reproductive health services), target Professionals (service providers at different levels), expected Outcomes (better sexual and reproductive health to increase quality of life), and Healthcare setting and context (all public, semi-private, and private centres providing services to breast cancer survivors including healthcare centres, offices, clinics, and hospitals). The main question of the study will be: what type of health services should survivors of breast cancer receive regarding prevention, screening, diagnosis, and management of their sexual and reproductive health problems?

Next, the guideline development core group will select recommendations regarding each question of the study based on their level of evidence, benefits (e.g. increasing quality of life, decreasing morbidity and number of referrals), harms (e.g. greater costs, increased morbidity and number of referrals), and their compatibility with local and cultural circumstances based on phase 1 and 2 results. The important outcomes will be selected based on the results of the qualitative study in phase 1 and review of the scientific evidence in phase 2. The outcomes that have a significant negative impact on patients’ physical, mental, and social well-being will be prioritized. Three techniques will be used: adoption (accepting existing recommendations from available guidelines as they are), adaptation (changing existing recommendations from available guidelines to meet local and cultural needs), and de novo development of recommendations if required (Iranian breast cancer survivors require a specific health service according to phase 1 results but there is no available recommendation in this regard in the literature based on phase 2 results) [18]. The strength of the recommendations will be determined according to Table 5.

**Table 5:** Grade of recommendation

Grade	Definition
A	All level 1 evidence shows that benefits are greater than harms
B	At least one piece of level 1 and level 2 evidence shows that benefits are greater than harms
C	There is no high-level evidence that benefits are greater than or equal to harms. Decisions should be made based on experts’ opinions
D	There is evidence that harms are greater than benefits

Adapted [38] from AACE protocol for standardized production of clinical practice guidelines [40]

In the next step, the guideline development core group will prepare a table including clinical questions with their corresponding recommendations indicating the source, level of evidence, grade of recommendation, benefits, and harms. This table, with the supporting scientific evidence, will be circulated among the scientific committee. We will ask the scientific committee to submit their opinions about each recommendation (agree, disagree, agree with some modifications) in written format. We will also ask them to inform us if they are aware of further scientific evidence. The guideline development core group will then modify the guideline based on the opinions of the scientific committee.

Subsequently, a meeting will be held including the guideline development core group, scientific committee, oversight committee, patient representatives, service providers, and stakeholders. Experts from the cancer department and department for standardizing clinical guidelines from the Ministry of Health and Medical Education in Iran will also attend this meeting. All participants will be informed in advance about the aim, scope, and process of guideline development orally and in writing. At the beginning of the meeting, SR will present a summary of the guideline development process, the opinions of the scientific committee, agreements, and disagreements. The participants will then discuss the recommendations considering everyone’s opinions including stakeholders. The executive considerations including acceptability, accessibility, and utilization of the health services will be discussed. After the meeting, the guideline development core group will modify the guideline based on the results of the meeting.

In the next step, another meeting will be held with the scientific committee. In this meeting, the nominal group technique (NGT) will be used to reach a consensus [31]. The recommendations with more than 80% agreement will be included in the guideline. For recommendations with less than 80% disagreement, the guideline development core group will decide and this will be mentioned in the guideline.

Finally, the guideline will be evaluated by internal and external reviewers using a quantitative approach. In the internal evaluation, the guideline development core group and scientific committee will evaluate the guideline using the AGREE II instrument. External evaluation will be performed in two steps. First, two Iranian experts in both guideline development and sexual and reproductive health will evaluate the guideline using the AGREE II instrument. Second, to evaluate the practicality of the developed guideline, we will circulate it among at least 30 service providers, asking them the last question in the AGREE II instrument as to whether they would recommend the use of the guideline. We will also ask them whether they have any suggestions for improvement. All suggestions and recommendations will be collected in written format. Subsequently, the comments and suggestions will be assessed by the guideline development core group and will be integrated into the final version of the guideline. The developed guideline will be updated every 3 years according to new scientific evidence.

## Discussion

This study was designed with the aim of developing and validating a clinical guideline on the sexual and reproductive health of breast cancer survivors in Iran. Breast cancer is the most prevalent cancer in Iran [2]. Age at breast cancer diagnosis is also significantly lower compared to the rest of the world, and many Iranian patients are still in their reproductive years [3]. In addition, studies have shown that Iranian breast cancer survivors do not receive sufficient information regarding reproductive health [32] and almost no information regarding sexual health [33]. Therefore, the development of this guideline will be extremely beneficial.

Some recommendations are not practical in different countries due to local, cultural, and organizational differences. The development and validation of new guidelines also requires substantial resources [30]. Therefore, here, we will use different techniques to avoid duplicating work efforts and to save limited available resources, while ensuring that the guideline meets the needs of Iranian breast cancer survivors. First, we will investigate the current situation regarding services that survivors of breast cancer in Iran receive for their sexual and reproductive health. We will explore the opinions of breast cancer survivors, their husbands, and service providers through a qualitative content analysis. Next, we will conduct a comprehensive literature review to find all available guidelines and recommendations in this regard worldwide. Then, we will use data from the qualitative study to modify currently available guidelines by adopting recommendations as they are or adapting them to local needs while also adding recommendations as required. The qualitative data will be collected from Tehran, which is the city with the highest population in Iran, and from referral centres with patients from around the country. Therefore, it will include views and experiences from a wide range of people. We will also use data from service providers at different levels and incorporate their ideas into the guideline to make it comprehensive and practical. The data will be collected from the public, semi-private, and private sectors. Therefore, the data set will include people from a wide socioeconomic range. To validate the guideline, we will use the AGREE II instrument and collect the opinions of experts from outside the study group, policy-makers, and future users in the field, as well as the experts within the study group. This design will improve the feasibility and practicability of the guideline.

In this study, we will use an exploratory sequential mixed methods approach. Although this approach is more complex and includes more steps, it will enable the development of a more valid and reliable guideline. A mixed methods approach and multiple techniques have often been used by other groups to develop guidelines and protocols. Schünemann et al. introduced the GRADE-ADOLOPMENT approach in 2017 to develop new guidelines by combining three techniques (adoption, adaptation, and de novo development of recommendations). They evaluated the recommendations using the GRADE framework. However, they did not use qualitative data [18]. In 2020, Salarvand et al. combined the results of a qualitative content analysis with these three techniques (adoption, adaptation, and de novo development of recommendations) to develop a comprehensive clinical practice guideline for cancer therapy-induced mucositis. They suggest this method as a practical approach to developing comprehensive clinical guidelines in developing countries [34]. Mixed methods approaches are currently being used by a number of Iranian study groups to develop multiple guidelines (e.g. sexual and reproductive health of surrogate mothers [35], perinatal palliative care for women with foetal anomaly diagnosis [36], and improving the childbirth experiences of Iranian women [37]).

Service providers can use the recommendations of this guideline at different levels in all centres providing services to breast cancer survivors, including hospitals, clinics, health centres, private offices, and palliative care and support centres.

During the process of breast cancer diagnosis and treatment, patients are often overwhelmed by multiple physical, mental, and social problems, and usually ignore their sexual and reproductive health issues. On the other hand, there are not enough experts in this field with sufficient knowledge and skills. Therefore, patients usually get little help in this regard. This guideline will provide a scientific and practical framework for providing sexual and reproductive health services for survivors of breast cancer in Iran, and can significantly improve patients’ quality of life. It can also be used as a model to develop guidelines for the sexual and reproductive health of survivors of the other kinds of cancer.

## Data Availability

The developed guideline will be available from the corresponding author on request.

## Abbreviations

AACE: American Association of Clinical Endocrinologists
ACS: American Cancer Society
AGREE II: Appraisal of Guidelines for Research and Evaluation II
AHRQ: Agency for Healthcare Research and Quality
ASCO: American Society of Clinical Oncology
BCY4: 4th International Consensus Conference for Breast Cancer in Young Women
CCO: Cancer Care Ontario
ESMO: European Society for Medical Oncology
ESO: European School of Oncology
GRADE: Grading of Recommendations Assessment, Development and Evaluation
NCCN: National Comprehensive Cancer Network
NGT: Nominal group technique
NICE: National Institute for Clinical Excellence
PIPOH: Population concerned, Interventions of interest, target Professionals, expected Outcome, and Healthcare setting and context
SIGN: Scottish Intercollegiate Guidelines Network
